# Venetoclax in combination with ponatinib for the treatment of asciminib-resistant chronic myeloid leukemia

**DOI:** 10.1038/s41375-025-02732-1

**Published:** 2025-08-26

**Authors:** Nikola Curik, Adam Laznicka, Jitka Krizkova, Pavla Suchankova, Adela Vavrova, Vaclava Polivkova, Eva Pokorna, Pavel Semerak, Pavel Burda, Daniela Kuzilkova, Tomas Kalina, Andreas Hochhaus, Katerina Machova Polakova

**Affiliations:** 1 Institute of Hematology and Blood Transfusion, Prague, Czech Republic; 2 Institute of Pathological Physiology, First Faculty of Medicine, Charles University, Prague, Czech Republic; 3 Second Faculty of Medicine, Charles University, Prague, Czech Republic; 4 CLIP (Childhood Leukemia Investigation Prague), Department of Pediatric Haematology and Oncology, Second Faculty of Medicine and Faculty Hospital Motol, Charles University, Prague, Czech Republic; 5 Abteilung Hämatologie/Onkologie, Klinik für Innere Medizin II, Jena University Hospital, Jena, Germany

**Keywords:** Chronic myeloid leukaemia, Chronic myeloid leukaemia

**TO THE EDITOR:**

Asciminib, an allosteric inhibitor specifically targeting the myristoyl pocket of BCR::ABL1, has expanded therapeutic options for heavily pretreated patients with chronic myeloid leukemia (CML), and for those with the BCR::ABL1 T315I mutation [1–3]. Several clinical studies evaluating asciminib in frontline CML therapy are currently ongoing (e.g., ASCI4FIRST, ASCI4START, ASCEND), with promising preliminary results indicating superior efficacy, improved tolerability, and a lower rate of treatment discontinuation due to toxicity compared to ATP-competitive inhibitors However, clinical trials of asciminib in both advanced-line and first-line CML treatment also report the emergence of resistance, often due to acquired or persisting mutations in *BCR::ABL1* [2, 4]. The full spectrum of these mutations remains unidentified, underscoring the need for further investigation and the exploration of backup therapeutic strategies, including combination therapies.

This EUTOS 2022 (European Treatment and Outcome Study) work sought to investigate *BCR::ABL1* mutations and other genetic alterations associated with asciminib resistance in CML myeloblastic clones and to assess the sensitivity of asciminib-resistant CML cells to treatment in vitr*o* and on a preclinical mouse model.

Established asciminib-resistant (ASCI-R) clones of KCL-22 cell line (*n* = 8; Supplementary Methods) were analyzed using Next Generation Sequencing (NGS) to identify somatic mutations in SH3, SH2, and kinase (KD) domains of *BCR::ABL1* and in 62 selected leukemia-associated genes [5]. 7/8 ASCI-R clones harbored mutations in the *KD BCR::ABL1* and 5/8 ASCI-R clones exhibited clone-specific mutations in other leukemia-associated genes including *ASXL1*, *EZH1*, *GATA2*, *NOTCH1*, *SF3B1*, *TET2*, *TP53*, and *ZRSR2* (Fig. 1A; Supplementary Table S1A), alongside with other mutations intrinsic to KCL-22 cells (Supplementary Table S1B). Mutations conferring resistance to asciminib were found at various regions of KD BCR::ABL1 including the myristoyl pocket (A337T – Clone D62; A337V - Clone B73; E509G – Clone C102; L510P – Clone C22), the contact site with the SH3 domain (K294E at Clones B91 and D31), the ATP binding site (F317L in Clone C102) and the α-helix at N-lobe (D276G in Clone C113). All ASCI-R clones exhibited overexpression of *BCR::ABL1*, which corresponds to the level of phosphorylated CRKL, a client molecule of BCR::ABL1 tyrosine kinase (Supplementary Figs. S1-S2).

**Fig. 1:**
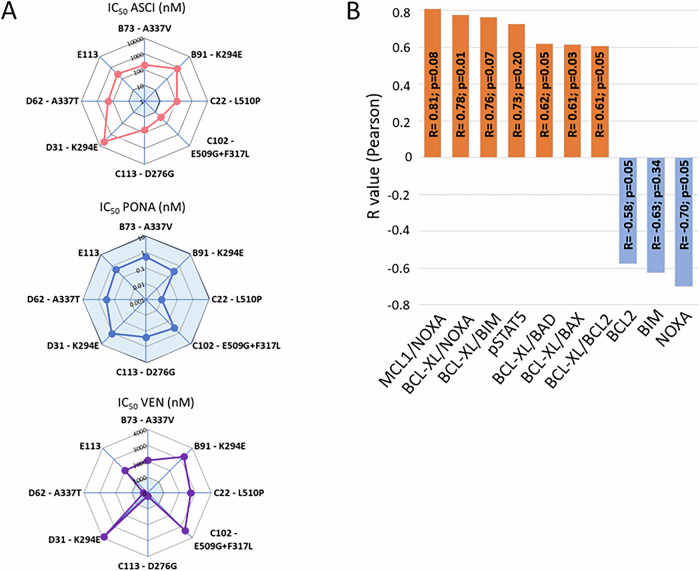
The sensitivity and resistance of ASCI-R clones to asciminib, ponatinib, and venetoclax in vitro. **A** Spider plots graphically display the IC_50_ values (nM) for asciminib (ASCI), ponatinib (PONA), and venetoclax (VEN) in ASCI-R clones after 48 h of treatment. The light blue marks the area of drug sensitivity. The IC_50_ numerical values (nM) for ASCI, PONA and VEN, respectively, are as follows: B73—199.25, 0.51 and 2060; B91—805.32, 0.33, and 3230; C22—108.62, 0.01, and 2710; C102—28.07, 0.36, and 3330; C113—65.44, 0.25, and 190; D31—4419, 1.16, and 3880; D62—205.23, 0.32, and 260; E113—271.7, 0.49, and 2010. **B** Correlation analysis shows strong correlations (R < −0.55 or R > 0.55) between protein levels or their mutual ratios in naïve ASCI-R clones and IC_50_ values for venetoclax. Positive and negative correlations are marked by orange and blue color, respectively. Significance is indicated by *p* values.

To determine impact of specific mutations in *KD BCR::ABL1* and leukemia-associated genes on asciminib resistance in our in vitro model of naturally mutated CML myeloblasts and to investigate treatment options including combinatory therapy in vivo, based on our previous results [5], IC_50_ values for asciminib, ponatinib, and venetoclax were assessed in each individual clones followed by 48 h of drug exposure. The level of resistance to asciminib (IC_50_ ≥ 10 nM) varied between clones. Also, the level of sensitivity to ponatinib (IC_50_ < 10 nM) varied, with IC_50_ values difference by up to two orders of magnitude (Fig. 1A). In vitro tests with venetoclax showed that 6/8 clones were resistant (IC_50_ ≥ 1000 nM) and 2/8 sensitive (IC_50_ < 1000 nM) to venetoclax (Fig. 1A). Both venetoclax-sensitive clones (C113 and D62) carried mutations in *ZRSR2* gene alongside BCR::ABL1 mutations (D276G and A337T, respectively) (Supplementary Table S1). Venetoclax, a BH3 domain mimetic, selectively binds to BCL2 and disrupts its ability to sequester and neutralize pro-apoptotic proteins. To further investigate the molecular determinants of venetoclax sensitivity and identify potential predictive protein markers in ASCI-resistant CML myeloblasts, we measured the levels of 16 proteins involved in apoptosis and pro-survival signaling using mass cytometry (CyTOF) (Supplementary Methods, Supplementary Table S2). BCL2 levels were increased and BCL-XL levels slightly decreased in venetoclax-sensitive clones compared to resistant clones, while no marked and consistent differences in the levels of other investigated proteins associated with sensitivity to venetoclax were observed (Supplementary Fig. S2). Cluster analysis, based on the levels of the 16 investigated proteins, partially separated venetoclax-naïve ASCI-R clones into distinct clusters according to their venetoclax sensitivity. The protein profile of the venetoclax-sensitive Clone C113 was notably different from that of other clones (Supplementary Fig. S3). A strong negative correlation (Rn<-0.55) was found between IC_50_ of venetoclax and levels of BCL2, BIM and NOXA in clones. Conversely, strong positive correlations (R > 0.55) were found between venetoclax IC_50_ and pSTAT5 levels, MCL1/NOXA levels ratio, and ratios of BCL-XL to various pro-apoptotic proteins. However, the level of significance *p* ≤ 0.05 was not reached for all observed correlations due to the small number of clones investigated and their uneven distribution between venetoclax-sensitive and venetoclax-resistant groups (Fig. 1B).

An equal number of cells from selected ASCI-R clones (*n* = 5), each harboring a unique BCR::ABL1 mutation (B73—A337V, B91—K294E, C22—L510P, C113—D276G, and D62—A337T) were used to establish a cell-line derived xenograft (CDX) model of polyclonal CML resistant to asciminib (Supplementary Methods). Mice were subcutaneously xenotransplanted by 5 × 10^6^ cells per mouse and randomly assigned into seven groups (*n* = 7 per group) according to the treatment method: (1) untreated controls (CTRL); (2) asciminib (ASCI) 30 mg/kg b.w.; (3) ponatinib (PONA) 25 mg/kg b.w.; (4) venetoclax (VEN) 50 mg/kg b.w.; (5) ASCI + PONA; (6) ASCI + VEN; (7) PONA + VEN. Therapeutical efficacy was assessed by regular monitoring of tumor growth with evaluation conducted on day 9 when first mouse in the control group was euthanized.

Among the treatment regimens, PONA, PONA + ASCI and PONA + VEN significantly suppressed tumor growth compared to the control (*p* < 0.001 each) (Fig. 2A). Moreover, PONA and PONA + VEN treatment regimens reduced tumors to the limit of measurability during the dosing period, with regression lasting until day 17 and day 24, respectively, when tumors relapsed (Fig. 2A). Tumor growth delay indices showed a significant delay in tumor growth for PONA and PONA + VEN treatment regimens (Supplementary Fig. S4). Notably, the PONA and PONA + VEN regimens significantly extended overall survival (OS), with median OS of 34.5 and 44 days, respectively, versus 12 days in the untreated group (*p* < 0.001) (Fig. 2B). Similarly, PONA and PONA + VEN significantly improved event-free survival (EFS; event defined as a tumor volume ≥500 mm^3^) (Supplementary Fig. S5). The combination of PONA + VEN significantly extended both OS and EFS compared to venetoclax alone (*p* < 0.001) and ponatinib alone (*p* < 0.01) (Supplementary Table S3). Expectedly, no survival benefit was found for PONA + ASCI combination over ponatinib monotherapy (Fig. 2B, Supplementary Fig. S5). Treatment toxicity was assessed by measuring the average relative weight loss of the mice at the time of treatment discontinuation compared to their baseline weight. PONA + VEN and PONA + ASCI treatments showed moderate toxicity (Supplementary Fig. S6). NGS analysis of cells from excised tumors revealed a predominant presence of Clone C22 (BCR::ABL1 L510P) in untreated tumors and tumors ineffectively treated with ASCI, VEN, and ASCI + VEN regimes. In contrast, tumors that relapsed after the cessation of effective treatment regimens were predominantly composed of Clone D62 (BCR::ABL1 A337T; IC_50_VEN = 260 nM, IC_50_PONA = 0.32 nM) in PONA + ASCI and PONA regimes, and Clone B91 (BCR::ABL1 K294E; IC_50_VEN = 3230 nM, IC_50_PONA = 0.33 nM) in PONA + VEN regime, suggesting their residual survival (Supplementary Fig. S7).

**Fig. 2:**
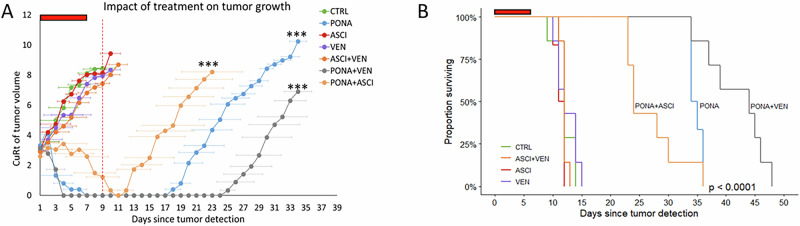
The impact of drug treatment on tumor growth in vivo and overall survival. **A** The average tumor volume was calculated by measurement of tumors growth in groups of mice according to treatment since the day of tumor detection. The values of Cube Root transformation (CuRt) are displayed. The error bars represent standard deviations of CuRt of tumor volumes. The tumor growth is shown until the first mouse was euthanized in the respective group. The dashed red line marks Day 9 as an evaluation time-point. The level of significance (type II ANOVA F test) is indicated: *** - *p* < 0.001. **B** Overall survival of mice according to the treatment groups. The level of significance (log-rank test) for impact of treatments regimens on survival rates is indicated. The red lines indicate the dosing period. CTRL control group without treatment, ASCI mice treated with asciminib, VEN mice treated with venetoclax, PONA mice treated with ponatinib, ASCI + VEN mice treated with asciminib and venetoclax, PONA + ASCI mice treated with ponatinib and asciminib; PONA + VEN mice treated with asciminib and venetoclax.

In summary, in our model, resistance to asciminib was primarily linked to KD BCR::ABL1 mutations, including recurrent mutations that emerge or persist under asciminib treatment (K294E, F317L, A337T, A337V) [2, 4, 6], and novel mutations identified in this study (E509G, L510P). The D276G mutation in the N-lobe region, associated with imatinib resistance [7], also conferred asciminib resistance. Only 1/8 ASCI-R clones (E113) exhibited BCR::ABL1-independent resistance. All ASCI-R clones were sensitive to ponatinib in vitro. Accordingly, experimental treatment in a preclinical CDX mouse model showed that ponatinib-based regimens effectively suppressed tumor growth and extended median survival. Sensitive clones to venetoclax treatment showed increased BCL2 levels, while venetoclax-resistant clones expressed high ratios of BLC-XL and MCL1 to pro-apoptotic proteins, particularly NOXA.

Increased BCL-XL and MCL1 levels, which can buffer pro-apoptotic proteins depleted by venetoclax, were linked to venetoclax resistance in various hematological malignancies [8]. These proteins are co-activated by BCR::ABL1-STAT5 signaling in CML [9, 10]. Indeed, high pSTAT5 levels positively correlated with venetoclax resistance in our study. Conversely, low and high levels of NOXA, which binds MCL1 to reduce its anti-apoptotic buffering potential, was associated with venetoclax resistance and sensitivity, respectively, in acute myeloid leukemia [8]. Additionally, mutations in the splicing factor gene *ZRSR2*, known to predict a good response to venetoclax in AML due to limiting MCL1 lifespan [8], were linked to venetoclax sensitivity in our study. Consistent with the above-outlined mechanisms, co-targeting BCR::ABL1 using ponatinib significantly enhanced the anti-tumor efficacy of venetoclax in vivo. The synergistic anti-leukemic effect of tyrosine kinase inhibitors combined with venetoclax has been previously demonstrated in both preclinical and clinical settings for BCR::ABL1-positive leukemias [11, 12]. Specifically, ponatinib-mediated inhibition of BCR::ABL1 and LYN kinases suppressed STAT5 phosphorylation and prevented upregulation of MCL1 in BCR::ABL1-positive lymphoblast cell lines [11]. Importantly, effective co-targeting of BCL2 and the BCR::ABL1–STAT5–MCL1/BCL-XL signaling axis allows for drug dose optimization, supporting a generally favorable safety profile in clinically relevant scenarios [12, 13]. However, ponatinib and venetoclax combination may lose efficacy when BCR::ABL1 activity is not adequately suppressed by ponatinib, e.g, in the case of CML resistance associated with BCR::ABL1 T315I-inclusive compound mutations [14, 15]. In such context, using low-dose ponatinib in combination with other drugs, notably asciminib and/or hydroxyurea, has been shown to yield additive or synergistic effects against CML blasts in preclinical models [5, 14].

## Supplementary information

Supplemental Material

## Data Availability

The datasets generated during and/or analyzed during this study are available from the corresponding author on reasonable request.
