# Supplementary material for: Venetoclax in combination with ponatinib for the treatment of asciminib-resistant chronic myeloid leukemia
Source: Leukemia. 2025 Aug 26;39(10):2555–8. doi: 10.1038/s41375-025-02732-1 (PMC12463652; doi:10.1038/s41375-025-02732-1)
Supplement: Supplementary file 1 — Supplemental Material [file 41375_2025_2732_MOESM1_ESM.pdf]

SUPPLEMENTARY METHODS

CML cell line and establishing asciminib-resistant (ASCI-R) clones

The KCL-22 cell line (ACC 519) was acquired from a publicly accessible biological resource center (the Leibniz Institute - Deutsche Sammlung von Mikroorganismen und Zellkulturen GmbH/DSMZ, Braunschweig, Germany). Mycoplasma contamination was tested no later than one month after the cells were thawed. Asciminib-resistant clones (ASCI-R; n=8) were established by single-cell FACS sorting (BD FACS Aria) into 96-well plates containing 0.01 nM asciminib, with a stepwise increase (one order of magnitude per month) up to 10 nM, as described previously.[1]

Next-generation sequencing (NGS)

The ASCI-R clones underwent characterization to identify somatic mutations in the *BCR::ABL1* SH3, SH2 and kinase domains and in other leukemia-related genes. *BCR::ABL1* amplicon libraries were prepared using the Nextera XT DNA Library Prep Kit (Cat. No. FC-131-1096, Illumina, San Diego, CA, USA). Data processing, error filtering, and mutation calling at significant levels were performed using the NextGene software (Softgenetics, State College, PA, USA) and the in-house bioinformatic tool NextDom.[2] Libraries for the DNA custom NGS panel of 62 genes frequently mutated in hematological malignancies,[3] were prepared using the SeqCap EZ HyperCap Workflow (Roche, San Diego, CA, USA) following the manufacturer's instructions. NGS libraries were sequenced on the MiSeq System (Illumina), and data evaluation was conducted using NextGene Software (SoftGenetics). The clinical relevance of the detected variants was assessed in the VarSome open-source database.[4] *BCR::ABL1* mRNA expression in ASCI-resistant clones was determined by RT-qPCR as (ratio of *BCR::ABL1* transcript copies to *GUSB* transcript copies)*100 (% IS).

Sensitivity of cells to ponatinib, asciminib and venetoclax *in vitro*, IC_{50} determination

$2x10^5$ cells of ASCI-R clones were cultivated in RPMI-1640 medium with asciminib, ponatinib or venetoclax solo in dilution series (0-1000 nM for asciminib; 0-100 nM for ponatinib; 0-10 µM for venetoclax) for 48 hours. Following cultivation, changes in proliferation for ASCI-R clones were measured in tetraplicate using the cell proliferation kit (WST-1 Assay Kit, Cat. No. ab65475, Abcam PLC, Cambridge, UK) with a spectrophotometer Synergy HT (BioTek, Winooski, VT, USA). IC_{50} values were calculated using freeware software AAT Bioquest (https://www.aatbio.com/tools/ic50-calculator).

Preparation of samples for mass cytometry

In-house antibodies conjugations with lanthanide and cadmium/platinum isotopes were performed using the Maxpar® X8 or MCP9 antibody labeling kits (Standard BioTools, South San Francisco, CA, USA), respectively, according to the manufacturer's instructions. Antibody cocktails (Supplementary Table S2) were prepared and handled as previously described.[5] Cells ($0.25x10^6$) were fixed in 1ml of freshly diluted 1.6% paraformaldehyde (10 min), washed and preserved at -20 °C in 10% glycerol in fetal bovine serum until staining day. The samples were thawed and washed with MaxPar Cell Staining Buffer (CSB; Standard BioTools). Barcoding was performed with 11 choose 1 strategy using either anti-CD45 or anti-HLA-I antibodies Briefly, barcoding antibodies were added (30 min, RT), cells were washed in CSB and pooled. To minimize technical variability, differently treated clones were barcoded and further processed in a single tube. To control for batch effects, a mixture of cell lines (KCL-22 and HBL2) was acquired together with barcoded healthy donor peripheral blood mononuclear cells as an internal spike-in control.[6] The samples were permeabilized with 80% ice-cold methanol for 30 minutes, then washed twice with CSB. A mixture of antibodies targeting proteins involved in apoptosis and cell signaling was added for 30 min (RT). Samples were washed twice, fixed with 1ml of freshly diluted 1.6% paraformaldehyde (10 min), and stored with Iridium and 10% glycerol at 4°C until acquisition day.

Mass Cytometry and data visualization

The instrument (CyTOF XT, Standard BioTools, CyTOF 8.1.0.18524 software) was prepared for acquisition following the manufacturer's recommendations. Samples were washed twice in CSB and twice in Cell Acquisition Solution (Standard BioTools), passed through a filter, and acquired at a maximum rate of 400 events per second. FCS files were processed using FlowJo (v10.5, FlowJo LLC). Cells were identified based on 191/193 Iridium staining, and individual samples were debarcoded manually with doublets excluded by removing illegal barcodes. The data were visualized as mean for median signal intensity from two independent measurements.

CDX model

For the establishment of the CDX model, female immunodeficient mice NOD.Cg-PrkdcscidIl2rgtm1Wjl/SzJ (n=49) aged 20-24 weeks were utilized. Number of animals per group (n=7) was calculated based on the estimated effect size of the drug on tumor growth, estimated variability (standard deviations), significance level and Power (0.9) using freeware software Experimental Design Assistant (Group and sample size | NC3Rs EDA). The number of animals was further adjusted in consideration of possible exclusions due to mouse injury during drug application or ineffective xenotransplantation. Mice were randomly and equally allocated to the control and experimental groups. Mice were weighed, and tumor sizes were measured daily with calipers. The treatment was administered orally once daily for 7 days from the onset of measurable tumors (≥ 5 mm in any direction). At the end of the experiment, tumor cells were isolated and analyzed using NGS to assess the representation of individual leukemic clones. Euthanasia criteria included: 1) tumor growth to 20 mm in one dimension; 2) visible physical or behavioral discomfort/skin necrosis. Moderate and severe toxicity thresholds were set at 10% and 20% weight loss, respectively. The assessment of drug effects on tumors and animals was unblinded. The experimental design was approved by the institutional Animal Care and Use Committee and Ministry of Education, Youth and Sports of the Czech Republic (approval number MSMT-5374/2024-4).

Statistical analyses

Comparative statistical tests, including Wilcoxon and t-tests, along with Pearson correlation analyses, were used to evaluate significant differences and correlations between venetoclax IC_{50} values and intracellular protein levels. Z-score normalization was applied to standardize protein level values across proteins, allowing for consistent comparison across samples. Heatmaps were generated with hierarchical clustering based on Euclidean distance to visually group samples with similar expression patterns. These analyses were performed using R-working web tool ClustVis2.0.[7] *In vivo* tumor growth was assessed using the type II ANOVA F test with the Kenward-Roger approximation for the denominators of degrees of freedom. Tumor growth curves among treatment groups were analyzed at a significance level of 95%. To enhance data visualization, a Cubic Root transformation (CuRt) of tumor volumes (e.g., [tumor volume (in mm^3)] ^⅓) was applied. Additionally, pairwise comparisons of tumor growth were conducted between treatment groups (SL 95%). The indexes of tumor growth delay incides (TGDIs) were calculated as a ratio of time to reach x-fold tumor volume increase in treated and untreated mice and analyzed for significance using a one-way ANOVA test as described by Guzik *et* al.[8] Survival probabilities and cumulative incidence rates were assessed using the Kaplan-Meier method. Survival and time-to-event were compared using the log-rank test. The comparison of risk ratios between groups was determined using the Cox proportional risk model at the significance level of 95%.

Large Language Models

The text of the manuscript has been edited with the assistance of the ChatGPT (OpenAI, 2024; https://chat.openai.com) to improve language clarity and grammar.

SUPPLEMENTARY REFERENCES

1. Curik N, Polivkova V, Burda P, Koblihova J, Laznicka A, Kalina T, *et al.* Somatic Mutations in Oncogenes Are in Chronic Myeloid Leukemia Acquired De Novo via Deregulated Base-Excision Repair and Alternative Non-Homologous End Joining. *Front Oncol* 2021; **11**: 744373

2. Benesova A, De Santis S, Polivkova V, Pecherkova P, Krizkova J, Suchankova P, et al. Unstable major molecular response as a trigger for next generation sequencing-based BCR::ABL1 mutation testing in chronic myeloid leukemia. *Am J Hematol* 2024; **99**: 759-762.

3. Curik N, Laznicka A, Polivkova V, Krizkova J, Pokorna E, Semerak P, et al. Combination Therapies with Ponatinib and Asciminib in a Preclinical Model of Chronic Myeloid Leukemia Blast Crisis with Compound Mutations. *Leukemia* 2024; **38**: 1415-1418.

4. Kopanos C, Tsiolkas V, Kouris A, Chapple CE, Albarca Aguilera M, Meyer R, et al. VarSome: the human genomic variant search engine. *Bioinformatics* 2019; **35**:1978-1980.

5. Schulz AR, Baumgart S, Schulze J, Urbicht M, Grützkau A, Mei HE. Stabilizing Antibody Cocktails for Mass Cytometry. *Cytometry A*. 2019; **95**: 910-916.

6. Kuzilková D, Bugarin C, Rejlova K, Schulz AR, Mei HE, Paganin M, et al. Either IL-7 activation of JAK-STAT or BEZ inhibition of PI3K-AKT-mTOR pathways dominates the single-cell phosphosignature of ex vivo treated pediatric T-cell acute lymphoblastic leukemia cells. *Haematologica* 2022; **107**:1293-1310.

7. Metsalu T and Vilo J. ClustVis: a web tool for visualizing clustering of multivariate data using Principal Component Analysis and heatmap. Nucleic Acids Res. 2015; **43(W1)**: W566-570. Available at: https://biit.cs.ut.ee/clustvis/

8. Guzik P, Benešová M, Ratz M, Monné Rodríguez JM, Deberle LM, Schibli R, et al. Preclinical evaluation of 5-methyltetrahydrofolate-based radioconjugates-new perspectives for folate receptor-targeted radionuclide therapy. *Eur J Nucl Med Mol Imaging* 2021; **48**: 972-983.

Supplementary Table S1

A

Clone	BCR::ABL1 mutations (transcript)	BCR::ABL1 mutations (HGVS)	Mutations in other leukemia-related genes (DNA custom panel)	Mutations in other leukemia-related genes (HGVS)
C22	L510P	L510P ABL1(NM_005157.6):c.1529T>C		
E113	-		**NOTCH1 Q862X**	NOTCH1(NM_017617.5):c.2584C>T
C102	E509G + F317L	E509G ABL1(NM_005157.6):c.1526A>G; F317L ABL1(NM_005157.6):c.951C>G		
D31	K294E	K294E ABL1(NM_005157.6):c.880A>G	**GATA2 A257D**; **EZH1 A279T**; ASXL1 S663R; TET2 P20Q	GATA2(NM_032638.5):c.770C>A; EZH1(NM_001991.5):c.844G>A; ASXL1(NM_015338.6):c.1989C>G; TET2(NM_001127208.3):c.59C>A
B73	A337V	A337V ABL1(NM_005157.6):c.1010C>T		
D62	A337T	A337T ABL1(NM_005157.6):c.1009G>A	**SF3B1 Q891R**; **TP53 M340V**; **ZRSR2 Y271C**	SF3B1(NM_012433.4):c.2672A>G; TP53 (NM_000546.6):c.1018A>G; ZRSR2(NM_005089.4):c.812A>G
B91	K294E	K294E ABL1(NM_005157.6):c.880A>G	**GATA2 R396Q**	GATA2(NM_032638.5):c.1187G>A
C113	D276G	D276G ABL1(NM_005157.6):c.827A>G	ASXL1 S663R; ZRSR2 H369L	ASXL1(NM_015338.6):c.1989C>G; ZRSR2(NM_005089.4):c.1106A>T

B

Clone	Mutations in other leukemia-related genes (DNA custom panel)	Mutations in other leukemia-related genes (HGVS)
C22, E113, C102, D31, B73, D62, B91, C113	DNMT3A E228K	DNMT3A(NM_022552.5):c.682G>A
	DNMT3A V227L	DNMT3A(NM_022552.5):c.679G>T
	SETD2 R1407Gfs*5	SETD2(ENST00000409792.4):c.4219del
	TET2 F868L	TET2(NM_001127208.3):c.2604T>G
	IKZF1 C150Y	IKZF1(NM_006060.6):c.449G>A
	SETD1B E1870K	SETD1B(NM_001353345.2):c.5608G>A
	PRPF8 R1402C	PRPF8(NM_006445.4):c.4204C>T
	TP53 P301Qfs*44	TP53(NM_000546.6):c.902del
	CEBPA H84Q	CEBPA(NM_004364.5):c.252C>G
	CEBPA H58Q	CEBPA(NM_004364.5):c.174C>A
	CEBPA C56W	CEBPA(NM_004364.5):c.168C>G
	CEBPA P39L	CEBPA(NM_004364.5):c.116C>T
	ASXL1 A654Gfs*4	ASXL1(NM_015338.6):c.1960dup
	ASXL1 P874L	ASXL1(NM_015338.6):c.2621C>T
	BCOR V878A	BCOR(NM_001123385.2):c.2633T>C

Supplementary Table S1. The mutational profiles of ASCI-R clones. (A) Clone-specific mutations identified in ASCI-R clones. Clones highlighted in yellow were used in xenotransplantation experiments as a pooled mixture. Mutations shown in bold represent variants of uncertain significance (VUS). Mutations highlighted in red were classified as likely pathogenic or pathogenic. The clinical relevance of all detected variants was evaluated using the VarSome open-source database. **(B)** Mutations detected in the parental KCL-22 cell line and shared across all derived ASCI-R clones. Variant annotations follow the Human Genome Variation Society (HGVS) nomenclature standards for DNA variants.

Supplementary Table S2

Marker	Clone	Metal isotope	Catalogue number	Vendor	Purpose	Conjugation
CD45	HI30	Y89	3089003B	Standard BioTools	barcode	In-house
CD45	MEM-28	106Cd	11-222-M001	Exbio	barcode	In-house
CD45	MEM-28	110Cd	11-222-M001	Exbio	barcode	In-house
CD45	MEM-28	111Cd	11-222-M001	Exbio	barcode	In-house
CD45	MEM-28	113Cd	11-222-M001	Exbio	barcode	In-house
HLA-I	W6/32	114Cd	BE0079	Bxcell	barcode	In-house
CD45	MEM-28	114Cd	11-222-M001	Exbio	barcode	In-house
HLA-I	W6/32	116Cd	BE0079	Bxcell	barcode	In-house
CD45	MEM-28	116Cd	11-222-M001	Exbio	barcode	In-house
CD45	MEM-28	194Pt	11-222-M001	Exbio	barcode	In-house
CD45	MEM-28	195Pt	11-222-M001	Exbio	barcode	In-house
CD45	MEM-28	196Pt	11-222-M001	Exbio	barcode	In-house
CD45	HI30	198Pt	3198001B	Standard BioTools	barcode	In-house
BCL-XL	54H6	142Nd	61060SF	Cell Signaling	panel - apoptosis	In-house
BIM	C34C5	145Nd	2933BF	Cell Signaling	panel - apoptosis	In-house
CRKL	Y244	148Nd	ab247210	Abcam	panel - signaling	In-house
pSTAT5 [Y694]	47	150Nd	3150005A	Standard BioTools	panel - signaling	Vendor
pAKT [S473]	D9E	152Sm	3152005A	Standard BioTools	panel - signaling	Vendor
BCL2	BCL-2/100	153Eu	11-668-C100	Exbio	panel - apoptosis	In-house
NOXA	114C307	156Gd	ab13654	Abcam	panel - apoptosis	In-house
BAX	2D2	158Gd	633602	BioLegend	panel - apoptosis	In-house
BID	Y8	159Tb	ab247217	Abcam	panel - apoptosis	In-house
pBAD [S136]	D25H8	161Dy	4366BF	Cell Signaling	panel - apoptosis	In-house
MCL1	Poly	163Dy	3163006A	Standard BioTools	panel - apoptosis	Vendor
BAD	Y208	165Ho	ab220116	Abcam	panel - apoptosis	In-house
BAK	G317-2	168Er	556382	BD	panel - apoptosis	In-house
CASPASE 3 (Cleaved,D175)	5A1E	170Er	9664BF	Cell Signaling	control - apoptosis	In-house
pERK1/2 [T202/Y204]	D13.14.4E	171Yb	3171010A	Standard BioTools	panel - signaling	Vendor
pCRKL [T207]	E9A1U	173Yb	94358SF	Cell Signaling	panel - signaling	Vendor
MYC	9E10	176Yb	3176012B	Standard BioTools	panel - apoptosis	Vendor

Supplementary Table S2. Antibodies used in mass cytometry (CyTOF) analysis.

Supplementary Table S3

A

Overall survival (OS)		
Treatment groups	Hazard ratio	P value
PONA vs CTRL	-Inf [-Inf;Inf]	0.0004
PONA + ASCI vs CTRL	-Inf [-Inf;Inf]	0.0001
PONA + ASCI vs PONA	2.706 [0.871;8.407]	0.0908
PONA + VEN vs CTRL	-Inf [-Inf;Inf]	0.0001
PONA + VEN vs PONA	0.081 [0.009;0.701]	0.0034
PONA + VEN vs VEN	-Inf [-Inf;Inf]	0.0001

B

Event-free survival (EFS)		
Treatment groups	Hazard ratio	P value
PONA vs CTRL	-Inf [-Inf;Inf]	0.0004
PONA + ASCI vs CTRL	-Inf [-Inf;Inf]	0.0001
PONA + ASCI vs PONA	4.028 [1.177;13.787]	0.0215
PONA + VEN vs CTRL	-Inf [-Inf;Inf]	0.0001
PONA + VEN vs PONA	0.110 [0.020;0.600]	0.0021
PONA + VEN vs VEN	-Inf [-Inf;Inf]	0.0001

Supplementary Table S3. The comparison of risk ratios for (A) death and (B) event, defined as tumor volume ≥500 mm^3. Relevant differences in groups were evaluated using the Cox proportional risk model at the significance level of 95%.

Supplementary Figure S1

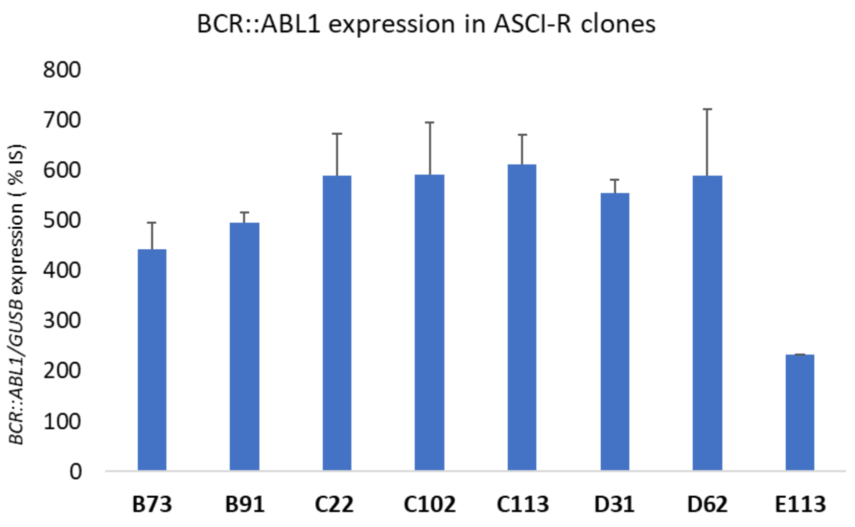

Supplementary Figure S1. The BCR::ABL1 mRNA expression in ASCI-R clones, normalized to GUSB, is represented by columns. The error bars represent standard deviations.

Supplementary Figure S2

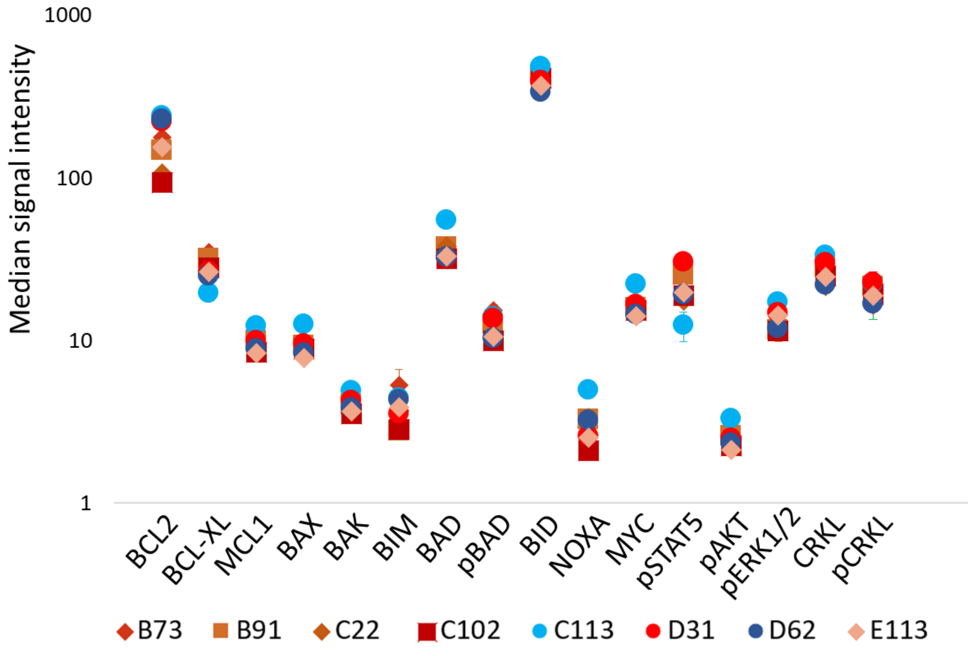

Supplementary Figure S2. Median signal intensity for 16 analyzed proteins, measured by mass cytometry in naïve ASCI-R clones. Resistance and sensitivity to venetoclax are indicated by red and blue, respectively. Values represent mean of medians from two independent experiments. Error bars represent standard deviations.

Supplementary Figure S3

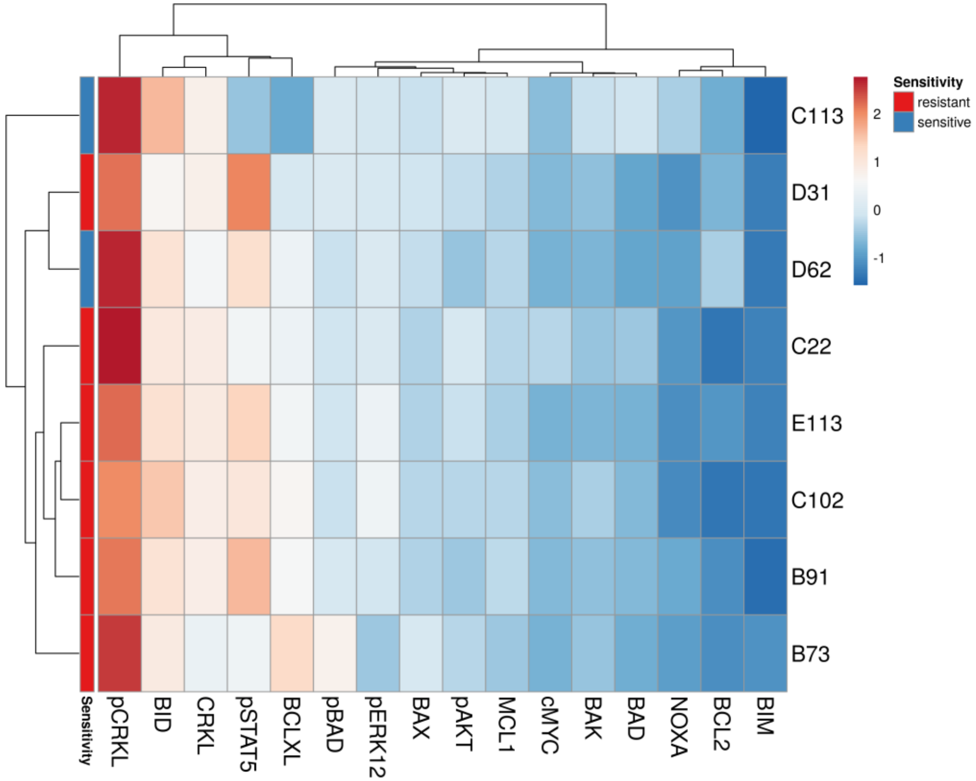

Supplementary Figure S3. Cluster analysis of ASCI-R clones based on their intracellular protein levels shown in association with their sensitivity or resistance to venetoclax. Resistance and sensitivity to venetoclax are indicated by red and blue color, respectively. Heatmap was generated with hierarchical complete-linkage clustering based on Euclidean distance.

Supplementary Figure S4

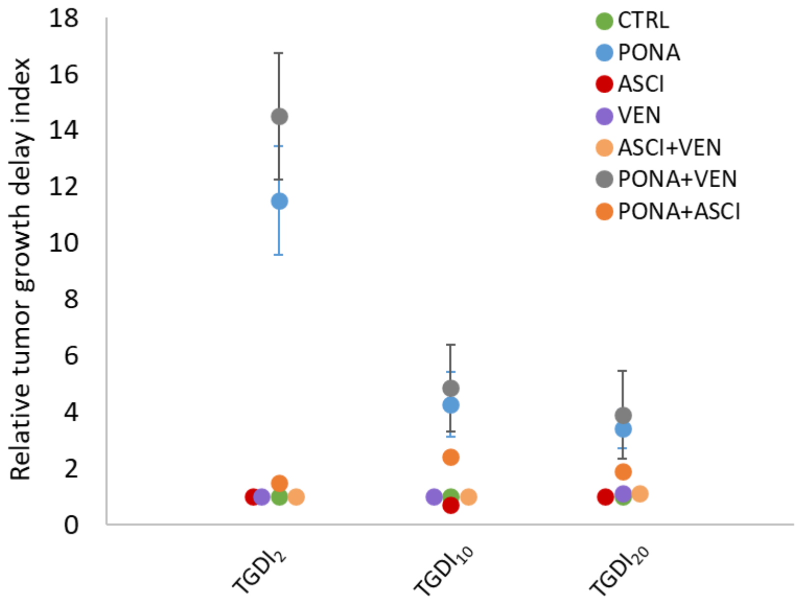

Supplementary Figure S4. The tumor growth delay incides (TGDIs) for 2-fold, 10-fold and 20-fold increase of original average tumor volume are displayed as indexes relatively to the growth of untreated tumors. The error bars represent standard deviations. CTRL – control group without treatment; ASCI – mice treated with asciminib; VEN – mice treated with venetoclax; PONA – mice treated with ponatinib; ASCI+VEN – mice treated with asciminib and venetoclax; PONA+ASCI – mice treated with ponatinib and asciminib; PONA+VEN – mice treated with asciminib and venetoclax.

Supplementary Figure S5

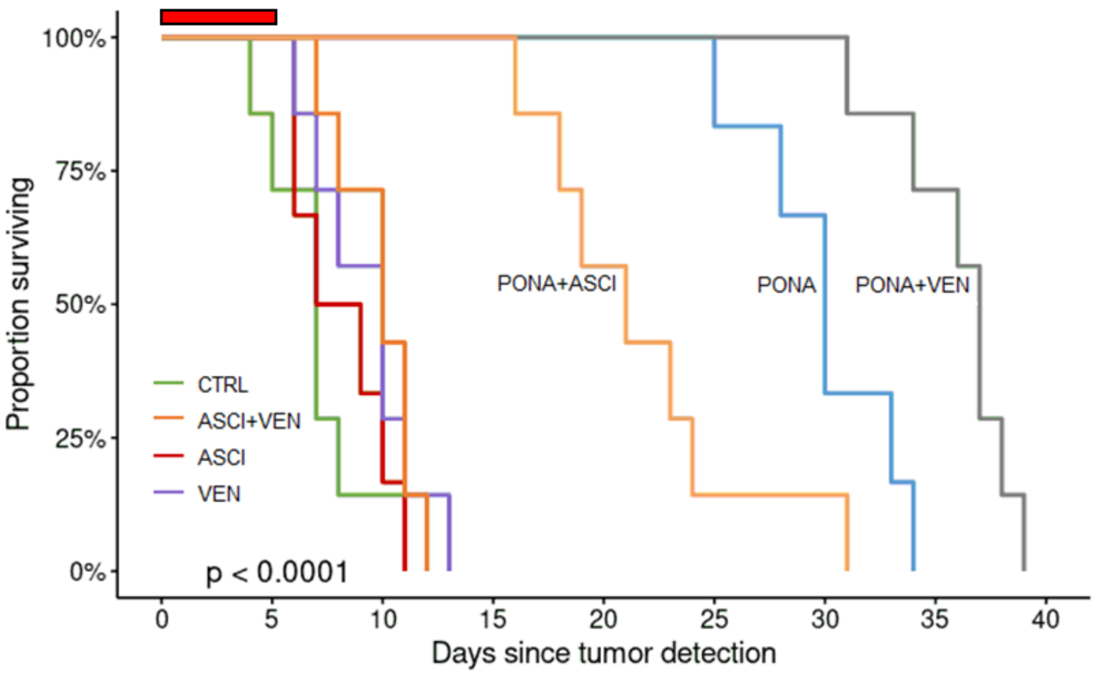

Supplementary Figure S5. Event-free survival of mice according to the treatment groups. Event was defined as tumor volume ≥500 mm3. The red line indicates the dosing period. CTRL – control group without treatment; ASCI – mice treated with asciminib; VEN – mice treated with venetoclax; PONA – mice treated with ponatinib; ASCI+VEN – mice treated with asciminib and venetoclax; PONA+ASCI – mice treated with ponatinib and asciminib; PONA+VEN – mice treated with asciminib and venetoclax.

Supplementary Figure S6

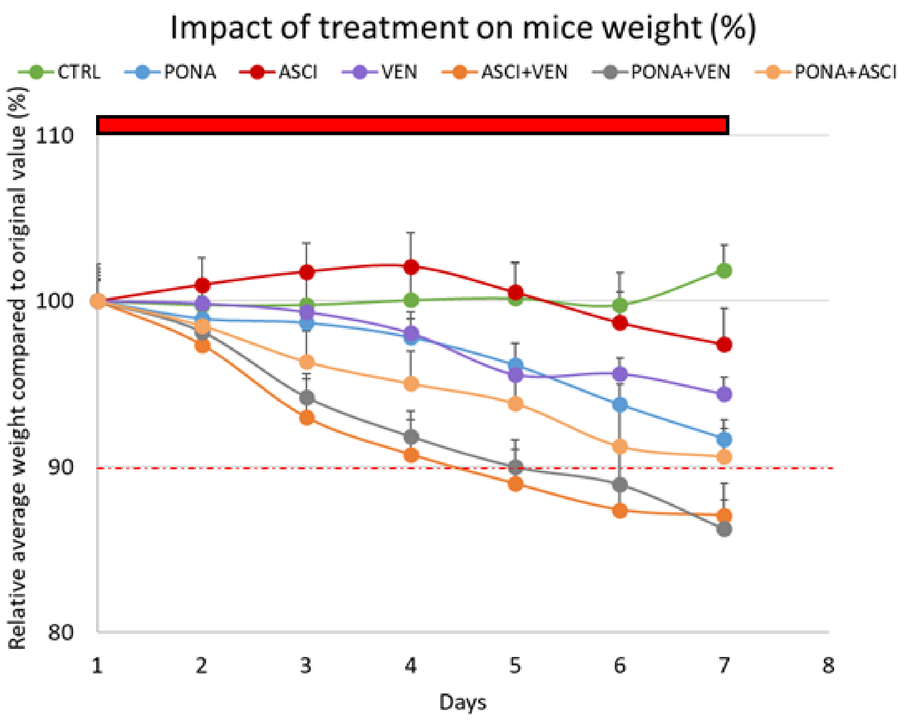

Supplementary Figure S6. The impact of treatment regimens on animal weight. The average body weight of mice during dosing period, is expressed as a percentage relative to the initial weight of the animals. The dashed red line marks of 10% weight loss, set as the level of mild toxicity. The error bars represent standard deviations. The red line indicates the dosing period. CTRL – control group without treatment; ASCI – mice treated with asciminib; VEN – mice treated with venetoclax; PONA – mice treated with ponatinib; ASCI+VEN – mice treated with asciminib and venetoclax; PONA+ASCI – mice treated with ponatinib and asciminib; PONA+VEN – mice treated with asciminib and venetoclax.

Supplementary Figure S7

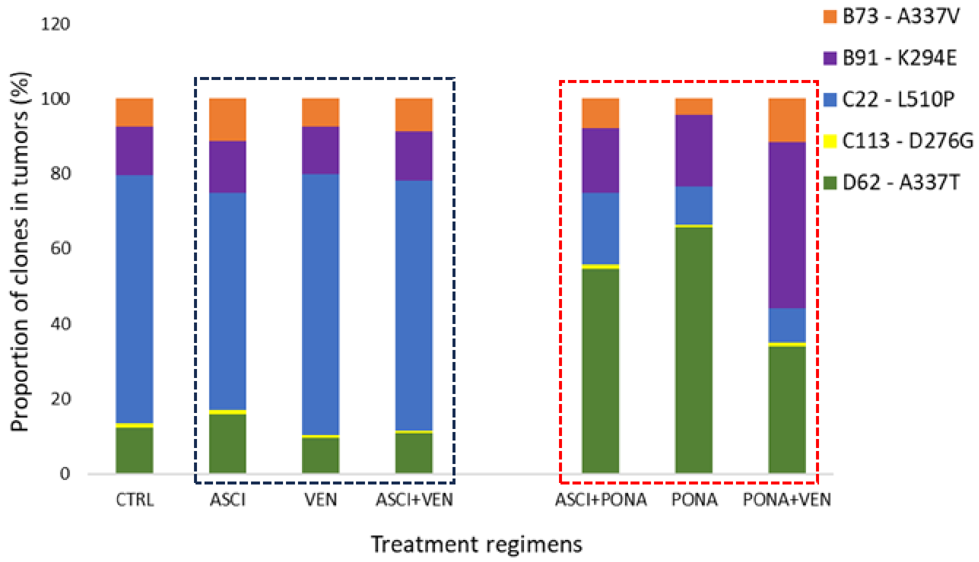

Supplementary Figure S7. The relative proportion of individual ASCI-R clones in tumors according to the treatment regimens is depicted. Ineffective and effective treatment regimes are marked by blue and red dashed line, respectively. CTRL – control group without treatment; ASCI – mice treated with asciminib; VEN – mice treated with venetoclax; PONA – mice treated with ponatinib; ASCI+VEN – mice treated with asciminib and venetoclax; PONA+ASCI – mice treated with ponatinib and asciminib; PONA+VEN – mice treated with asciminib and venetoclax.
